# Supplementary material for: A Non-Invasive, Label-Free Method for Examining Tardigrade Anatomy Using Holotomography
Source: Tomography. 2025 Mar 14;11(3):34. doi: 10.3390/tomography11030034 (PMC11946113; doi:10.3390/tomography11030034)
Supplement: Supplementary file 1 [file tomography-11-00034-s001.zip › tomography-3466833-supplementary-Table S1, Figures S1-S4.pdf]

Table S1. Tomocube HT-X1 Holotomography System—key performance specifications. Information from the manufacturer's website (Tomocube).

Parameter	Specification / Details
Objective lens	40x NA 0.95 (air)
Light source	Low coherent single-beam white LED (450nm for HT)
Fluorescence excitation/bandwidth	378/52, 474/27, 554/23, 635/18 nm
Lateral Resolution	156/161/179/205 nm
Axial Resolution	1069/1211/1387/1695 nm
3D Field of View	218 μm x 165 μm
Temporal resolution	6.5 seconds/image
Depth of field	Max. 146 μm

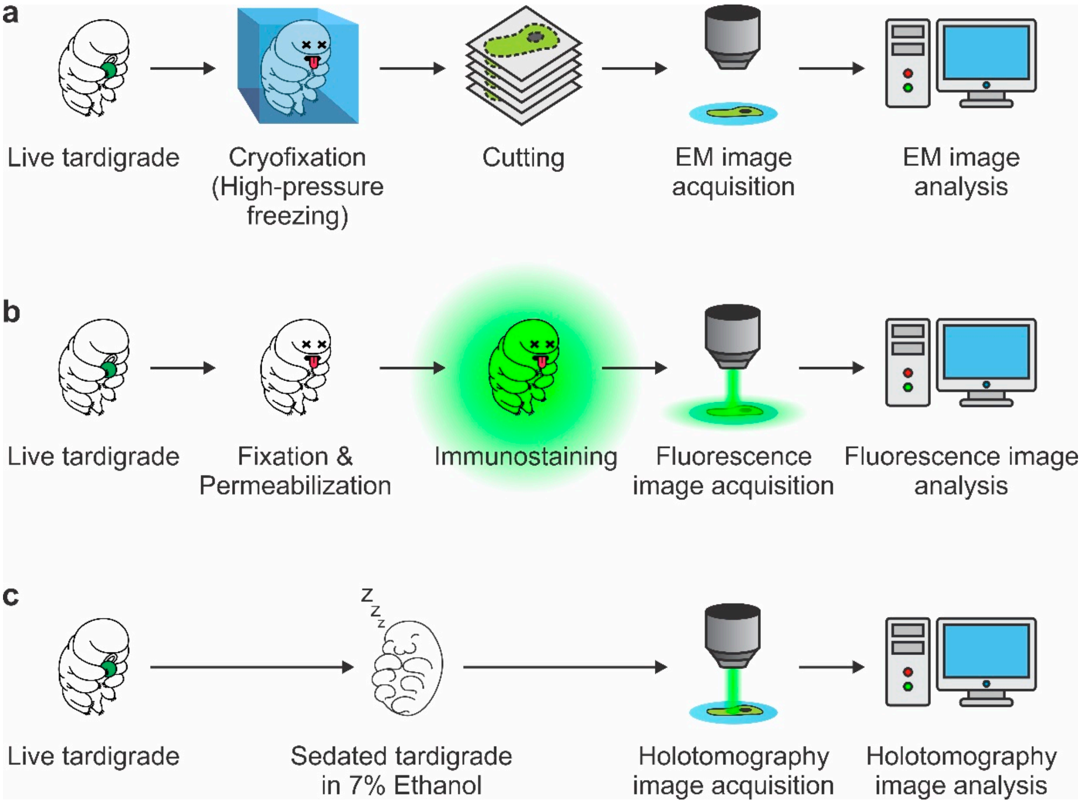

Figure S1. Visual comparison of tardigrade imaging modalities. **a.** Electron microscopy (EM): A live tardigrade undergoes high-pressure freezing (HPF) for cryofixation, followed by ultrathin sectioning to prepare the sample for imaging. The electron microscopy imaging process is then performed, and the resulting data undergo detailed EM image analysis to extract structural information**. b.** Fluorescence imaging: A live tardigrade is subjected to fixation and permeabilization to preserve cellular structures and allow for antibody penetration. Immunostaining is then performed to label specific proteins or cellular components with fluorescent markers. The fluorescence imaging system captures the stained structures, and the acquired images are subsequently analyzed to study the distribution and localization of targeted biomolecules**. c.** Holotomography: In this technique, a live tardigrade is first sedated using 7% ethanol to minimize movement artifacts. The specimen is then imaged using holotomography, a label-free quantitative phase imaging technique that reconstructs 3D refractive index distributions. Finally, the acquired holotomography images are reconstructed to assess internal structures and cellular morphology.

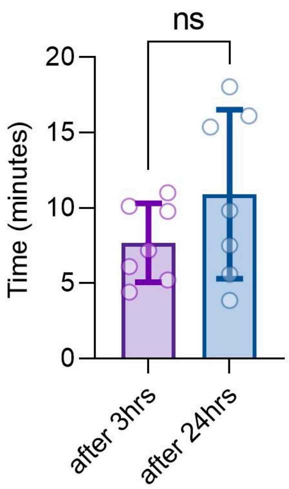

Figure S2. Recovery time of tardigrades after 3-hour and 24-hour incubation in 7% ethanol. Bar chart showing the recovery time (in minutes) of tardigrades (n = 7) after 3-hour and 24-hour incubation in 7% ethanol. Statistical analysis was performed using a two-sided t-test, P = 0.1943 (ns: not significant). Error bars represent the standard deviation.

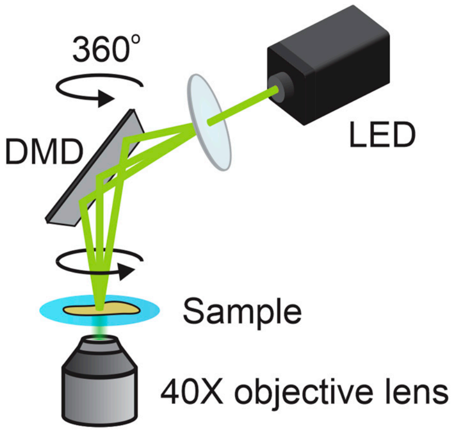

Figure S3. Schematic of holotomography principle. Holotomography employs an LED beam to measure the three-dimensional refractive index (RI) distribution of cells. The system captures multiple two-dimensional holograms of a sample at various illumination angles, which are then used to reconstruct a 3D RI tomogram through an inverse scattering algorithm. Tomocube enhances this process with highly precise laser beam control, enabled by digital micromirror device (DMD) technology (Texas Instruments).

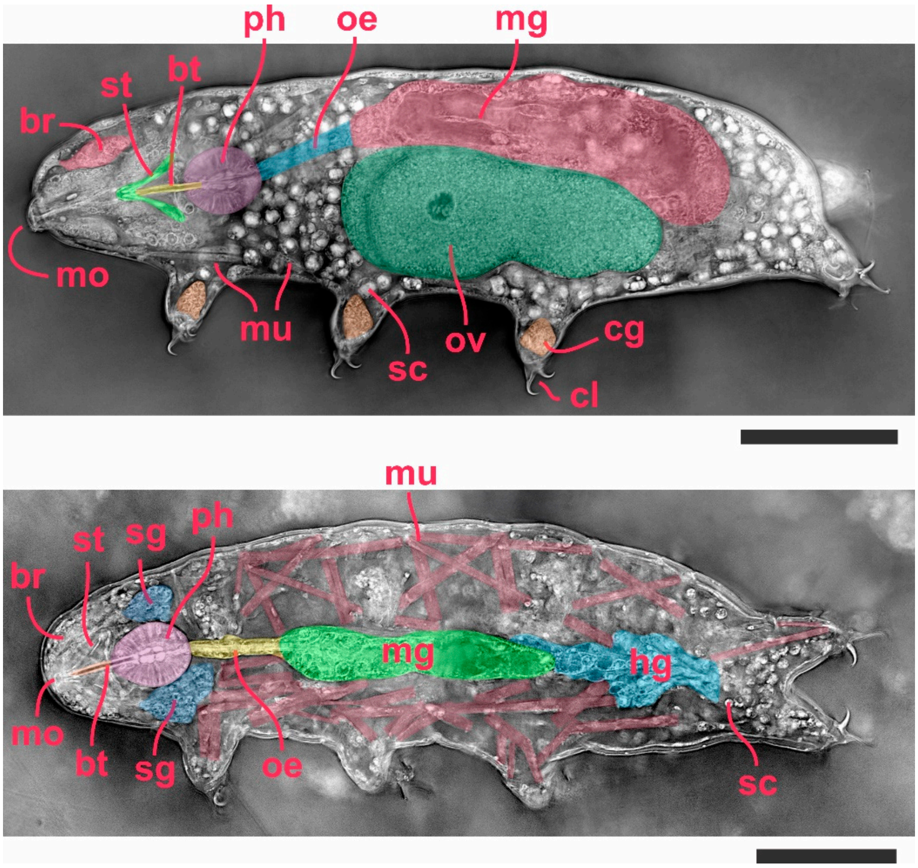

Figure S4. Pseudo-colored holotomographic image highlighting multiple organs of the tardigrade. Scale bar: 50 μm. Abbreviations: br—brain; st—stylet; bt—buccal tube; ph—pharynx; oe—esophagus; mg—midgut; mg—hindgut; mo—mouth; mu—muscle; sc—storage cells; sg—saliva glands; ov—ovary; cl—claws; cg—claw glands.

Video S1. 3D Render of Tardigrade Holotomography—Oscillatory Perspective. The video was compressed using an MPEG-4 H.264 standard.

Video S2. 3D Render of Tardigrade Holotomography—360° Orbiting View. The video was compressed using an MPEG-4 H.264 standard.
